# Ginkgolide B Protects Against Ischemic Stroke *via* Targeting AMPK/PINK1

**DOI:** 10.3389/fphar.2022.941094

**Published:** 2022-06-28

**Authors:** Yile Cao, Lei Yang, Hong Cheng

**Affiliations:** ^1^ Department of Clinical Medicine, School of Medicine, Yangzhou University, Yangzhou, China; ^2^ Department of Orthopedics, Taizhou People’s Hospital, Taizhou, China; ^3^ Medical College, Yangzhou University, Yangzhou, China

**Keywords:** stroke, apoptosis, AMPK, PINK1, OGD, t-MCAO, ginkgolide B

## Abstract

**Introduction:** Ginkgolide B (GB), which is an active constituent derived from Ginkgo biloba leaves, has been reported to ameliorate Alzheimer’s disease (AD), ischemic stroke, as well as other neurodegenerative diseases due to its viable immunosuppressive and anti-inflammatory functions. However, it has yet to be proven whether GB inhibits neuronal apoptosis in ischemic stroke.

**Methods:** In the present research, the inhibition function of GB on neuronal apoptosis and its underpinning process(s) after cerebral ischemia were studied through transient middle cerebral artery occlusion (t-MCAO) in an *in vivo* rat model as well as in cultured SH-SY5Y cells subjected to oxygen and glucose deprivation (OGD)/reoxygenation *in vitro*. The neurological score was calculated and Nissl and TUNEL staining were performed to evaluate the stroke outcome, neuronal loss, and neuronal apoptosis. Subsequently, the western blot was utilized to detect Bcl2 and p-AMPK/AMPK expression.

**Results:** Compared to t-MCAO rats, rats receiving GB treatment showed a significant reduction of neuronal loss and apoptosis and improved neurological behavior at 72 h after MCAO. GB treatment also upregulated the expression of Bcl2 and p-AMPK. *In vitro*, GB suppressed the apoptosis in OGD/reoxygenation-challenged neuronal SH-SY5Y cells through AMPK activation.

**Conclusions:** Our observations suggest that GB enhanced AMPK activation in neural cells, reducing neuronal apoptosis, thus eventually preventing ischemic stroke.

## Introduction

Ischemic stroke is a kind of brain damage induced by a rapid reduction in cerebral blood flow (CBF) and a subsequent shortage of oxygen and glucose in the blood supply to the brain (Siniscalchi et al., 2014). The impairment caused by ischemia to the brain could be partly restored if the patient receives suitable therapy with thrombolytics during a specified time limit (about 3–4.5 h) (Drieu et al., 2018). Although effective, thrombolytics, including tissue-type plasminogen activator (t-PA), are restricted in their use due to their adverse impacts and relatively limited treatment timeframe (Zhang et al., 2005; Khan et al., 2007). Therefore, finding a successful stroke treatment method is in urgent need in clinical practice (Amani et al., 2019).

Both mitochondrial function and the glucose metabolic pathway are combined with redox signaling and are remarkably responsive to cerebral ischemia. It is the redox signaling system that regulates the generation of mitochondrial oxidant hyperoxia under normal physiological states (Hu et al., 2016; Yang and Long, 2018; Chen et al., 2019). Disturbances in glucose metabolism result in cerebral ischemia damaging mitochondrial transfer proteins (depolarized mitochondria), resulting in the presence and progression of hydrogen peroxide and superoxide at an unregulated rate (Akhmedov et al., 2015; Yang et al., 2019; Zuurbier et al., 2020). Eventually, these factors contribute to neuronal apoptosis.

PINK1 is a mitochondrial threonine/serine kinase stably located on the outer membrane of mitochondria that have suffered from damage. The substrates of PINK1 include the E3 ubiquitin ligase Parkin and ubiquitin itself. The phosphorylation of Parkin and ubiquitin cause the unnecessary depolarization of mitochondria, ubiquitinating them for selective degradation (Kim et al., 2008; Kane et al., 2014; Koyano et al., 2014). Therefore, PINK1 has an important role in mitochondria quality control. In addition, some studies have also shown that the mitogen-activated protein kinase (MAPK) pathway is essential for PINK1-dependent neuroprotection (Hang et al., 2015; Cao et al., 2020; Seabright and Lai, 2020).

Ginkgolide B (GB) isolated from *Ginkgo biloba* leaves was revealed to have anti-inflammation and antioxidant effects in ischemic stroke (Chi et al., 2015; Shu et al., 2016; Feng et al., 2019; Liu et al., 2019). There is evidence that GB ameliorates oxidative stress responses related to AMPK activation (Jiang et al., 2020; Wang et al., 2021). However, whether the activation of AMPK caused by GB treatment can protect mitochondrial function and attenuate neuronal apoptosis during stroke remains uncertain.

## Materials and Methods

### Animals and Drug Treatment

The APExBIO Technology LLC (Houston, TX, United States) supplied Ginkgolide B (Cas No. 15291-77-7) that was dissolved in DMSO and had a 99.5 percent purity. Sprague Dawley (SD) male rats (220–300 g) (Beijing Vital River Laboratory Animal Technology Co., Ltd., Beijing, China) were utilized in the present research. The rats were kept in standard conditions (12 h darkness-light light-dark cycle and ambient temperature at 23 ± 2°C). Then, all rats were housed for at least 1 week before surgery. Subsequently, the rats were classified at random into four experimental cohorts (*n* = 10 for each cohort), namely MCAO cohort, Sham-treated cohort, GB-treated cohort, and GB treated/MCAO cohort. The operation for Middle Cerebral Artery Occlusion (MCAO) was carried out in the same manner as discussed previously (Bai et al., 2016). Specifically, the rats were placed in a supine posture after being anesthetized using isoflurane. Then, the internal carotid artery (ICA), external carotid artery (ECA), and right common carotid artery (CCA) were dissected *via* a midline neck cut. The MCA was subjected to blocking by introducing an intraluminal silicone coating filament (Cinon Tech Co. Ltd., Beijing, China) into the CCA lumen and gradually advancing it into the ICA until it reached a location about 18 mm distal to the branching of the carotid artery. The filament was kept intact for 90 min before being slowly drawn back for a short duration to enable the restoration of blood flow in anticipation of reperfusion. The sham cohort was subsequently subjected to the identical experimental protocol, with the exception of the filament placement. One hour following surgery, GB (4 mg/kg) was administered intraperitoneally to the rats twice per day for three consecutive days following MCAO. A total of 10 rats died as a result of the surgical operations, with 5 from the MCAO cohort and another 5 from the GB treated/MCAO cohort dying during the process. An equivalent volume of saline instead of GB was injected into the rats from the sham and MCAO cohorts. The surgical operations led to the deaths of eight rats, with four from the MCAO cohort and another four from the MCAO + GB cohort. Once 72 h had elapsed following reperfusion, the rats were subjected to decapitation and their brains were harvested for analysis. In accordance with the National Institutes of Health’s Guide for the Care and Use of Laboratory Animals, all protocols utilized in the present research were subjected to approval by the Ethics Committee for the Use of Experimental Animals of Jiangsu Kanion Pharmaceutical Co. Ltd. State Key Laboratory of New Pharmaceutical Process for Traditional Chinese Medicine.

### Neurobehavioral Testing

A composite neuro score of 28 points was utilized to assess the sensory and motor capabilities of the rats receiving MCAO (Encarnacion et al., 2011). Specifically, this neural score was comprised of 11 assessments with a total combined score of 28, where a total score of 0 denoted significant neurological dysfunction, whereas a total score of 28 showed normal function without neurological impairment. The 11 tests were listed below (Siniscalchi et al., 2014) circling (maximum 4 points), (Drieu et al., 2018) motility (maximum 3 points), (Zhang et al., 2005) general condition (maximum 3 points), (Khan et al., 2007) righting reflex when placed on back (maximum 1 point), (Amani et al., 2019) paw placement of each paw onto a table top (maximum 4 points), (Hu et al., 2016) ability to pull self up on a horizontal bar (maximum 3 points), (Yang and Long, 2018) climbing on an inclined platform (maximum 3 points), (Chen et al., 2019) grip strength (maximum 2 points), (Akhmedov et al., 2015) contralateral reflex (maximum 1 point) and (Yang et al., 2019) contralateral rotation when held by the base of tail (maximum 2 points), and (Zuurbier et al., 2020) visual forepaw reaching (maximum 2 points).

### Nissl Staining

The brains of rats from each respective cohort were harvested, followed by fixing in a 10% buffered neutral formalin solution. After standard paraffin embedding, cresyl violet (Nissl staining) was utilized to stain segments. Specifically, dehydration of the sections was performed utilizing increasing concentrations of ethanol in water (30, 50, 75, 95, and 100%), followed by rehydration using decreasing concentrations of the same solution (95, 75, 50, and 30%). Subsequently, the sections were subjected to staining for 30 min utilizing cresyl violet solution (0.25 percent) at ambient temperature, followed by stepwise dehydration with 95 and 100% ethanol in water, and clearing using xylene. A fluorescent microscope (Nikon Eclipse 80i, Tokyo, Japan) was utilized to capture microscopy pictures for the purpose of detecting the neuronal loss and the images were subsequently evaluated utilizing NIS-Elements D (version 5.0). Enumeration of the number of neurons was performed from the ipsilateral and contralateral sides of the cerebral cortex and striatum under x400 magnification (Miyajima et al., 2018). Six rats were used to acquire data for two fields in each section.

### TdT-Mediated dUTP Nick End Labeling Staining

To determine neuronal apoptosis, TUNEL (TdT-mediated dUTP nick end labeling) staining was performed utilizing a Roche *in Situ* Cell Death Detection Kit in accordance with the guidelines stipulated by the manufacturer. Microscopy images were obtained using a fluorescence microscope (Nikon, Tokyo, Japan) and were evaluated utilizing the NIS-Elements D (version 5.0). Enumeration of the number of positive cells was performed from the ipsilateral and contralateral sides of the cerebral cortex and striatum under x400 magnification. Six rats were used to acquire data for two fields in each section.

### Cell Culture

The Cell Bank of the Chinese Academy of Sciences (no. CRL-2266) supplied the SH-SY5Y cells, which were used to culture a high glucose DMEM (Thermo Fisher Scientific, Waltham, MA, United States) comprising 10 percent fetal bovine serum (FBS) (Thermo Fisher Scientific, Waltham, MA, United States) and 1% penicillin-streptomycin. Subsequently, the cell culture media was replenished every 2 days. The cells were kept at 37°C in a humid chamber containing 5 percent CO_2_

### Hypoxia/Reoxygenation

The cells were exposed to H/R as previously reported (Miglio et al., 2004) after being treated for 6 h in the presence or absence of 100 μM GB (Liu et al., 2019). Briefly, the culture media was substituted with OGD buffer (pH 7.4; 2.3 mM CaCl_2_; 3.6 mM NaHCO_3_; 5.0 mM HEPES; 5.6 mM KCl; and 154 mM NaCl) and was then put into an airtight chamber where the samples were gassed for 10 min with 95% N_2_–5% CO_2_. The compartment was completely sealed, which was then moved to a humified incubator at 37°C containing 5 percent CO_2_ for 16 h 24 h prior to subsequent experiments, the chamber was opened to initiate reoxygenation and restore the cells to their previous normal culture states.

### Transient Transfection With Small Interfering RNA

SH-SY5Y cells were subjected to transient transfection with 100 nM siRNAs targeting AMPKα1/α2 (sc-29673 and sc-38923) or non-silencing control siRNA (scram) (sc-37007) (Santa Cruz Biotechnology, Santa Cruz, CA, United States) for the purpose of knocking down endogenous AMPK. Lipofectamine™ RNAiMAX (Thermo Fisher Scientific, Waltham, MA, United States) was employed as the transfection agent, and the cells were maintained in a culture media in the absence of antibiotics in accordance with the guidelines stipulated by the manufacturer.

### Cellular Viability Assessment

As noted earlier, Taveira et al. (2014) the viability of cells was determined using an MTT assay. An ELISA microplate reader (Molecular Devices, Sunnyvale, CA, United States) was utilized for the purpose of measuring the absorption spectrum of produced formazan crystals at 570 nm. The findings are then presented as a percentage of the obtained control cell values.

### Hoechst 33342 Staining

Seeding of the SH-SY5Y cells was performed at a density of 2×10^5^ cells/well in 6-well plates. After conducting various forms of treatment, the cells were rinsed using PBS before loading them with Hoechst 33342 dye (10 µg/ml, Sigma-Aldrich, St. Louis, MO, United States) for 15 min. Images were obtained using a fluorescence microscope (Nikon, Tokyo, Japan) and were evaluated utilizing NIS-Elements D (version 5.0). Subsequently, the isolated nuclei were examined at a magnification of x400 to differentiate the typical homogeneous nuclear pattern from the apoptotic cells’ compacted merged chromatin pattern. With respect to the quantification of cell apoptosis, 200 nuclei were examined from six randomly chosen microscopic views. The total number of apoptotic cells in each section was derived and presented as the percentage of the total cell number. Three individual sections were evaluated.

### Mitochondrial Membrane Potential Measurement

A mitochondrial membrane potential assay kit (C2006, Beyotime Biotechnology, Shanghai, China) was utilized to measure the MMP of cells. After treatment, SH-SY5Y cells were subjected to incubation for 20 min in darkness with a JC-1 staining solution. Thus, ΔΨm was expressed by the ratio of green (monomeric form, demonstrating depolarized/low MMP) over red (aggregated form, signifying polarized/normal MMP) determined with the aid of the Becton-Dickinson FACS Calibur system (BD Biosciences, Franklin Lakes, NJ, United States).

### Immunofluorescence Staining

To evaluate the expression of PINK1 in the outer mitochondrial membrane, incubation of the SH-SY5Y cells was performed in a glass chamber slide (Thermo Fisher Scientific, Waltham, MA, United States) and treated with or without GB under hypoxia/reoxygenation conditions. Subsequently, the cells were subjected to 15 min of fixing using 4 percent paraformaldehyde at ambient temperature, followed by permeabilization of the cells using 0.1 percent Triton X-100 and blocking using 10 percent goat serum. Then, the cells were subjected to incubation with anti-PINK1 (23274-1-AP, 1:200, Proteintech, Wuhan, China) and 488-conjugated mouse anti-Tom 20 antibody (sc-17764 AF488, 1:100, Santa Cruz Biotechnology, Santa Cruz, CA, United States) over the night at a temperature of 4°C and 594-conjugated donkey anti-rabbit antibody (Thermo Fisher Scientific, Waltham, MA, United States) at ambient temperature for 1 h. Eventually, the slides were mounted, followed by imaging with the aid of a fluorescence microscope (Nikon, Tokyo, Japan).

### Western Blotting

Protein concentrations obtained from the infarcted brain tissues or SH-SY5Y cells were assessed utilizing a BCA Protein Assay Kit (KeyGEN BioTECH, Nanjing, China). Specifically, proteins in identical quantities were treated to sodium dodecyl sulfate-polyacrylamide gel electrophoresis (SDS-PAGE) with 30 ug proteins added into per lane for the electrophoresis, followed by loading onto polyvinylidene fluoride (PVDF) membranes (Millipore, Billerica, MA, United States). Once the membranes had been blocked, incubation of the membranes using primary antibodies at 4°C throughout the night was performed, followed by another incubation using HRP-conjugated secondary antibodies at ambient temperature for 1 h. The membranes were then exposed and analyzed utilizing an ECL system (Tanon, Shanghai, China). The following were the primary antibodies that were employed in the protocol: anti-AMPK (ab32047), p-AMPK (ab133448), PINK1 (ab186303), Bcl-2 (ab196495), and Bax (ab32503). The secondary antibody used was an HRP-conjugated anti-GAPDH monoclonal antibody (HRP-60004, 1:5000, Proteintech, Rosemont, IL, United States).

### Statistical Analysis

The findings were analyzed using the Statistical Product and Service Solutions (SPSS) software (version: 22.0) (IBM, Armonk, NY, United States) and data were expressed as the means ± SEM. When comparing the different experimental cohorts, one-way ANOVA was utilized and Tukey’s *post hoc* analysis was performed for multiple range tests. Statistical significance was deemed to have been attained at *p* < 0.05.

## Results

### Ginkgolide B Treatment Improved the Composite Neuro Score and Attenuated Neuronal Loss and Apoptosis in Rats After Middle Cerebral Artery Occlusion

**FIGURE 1:**
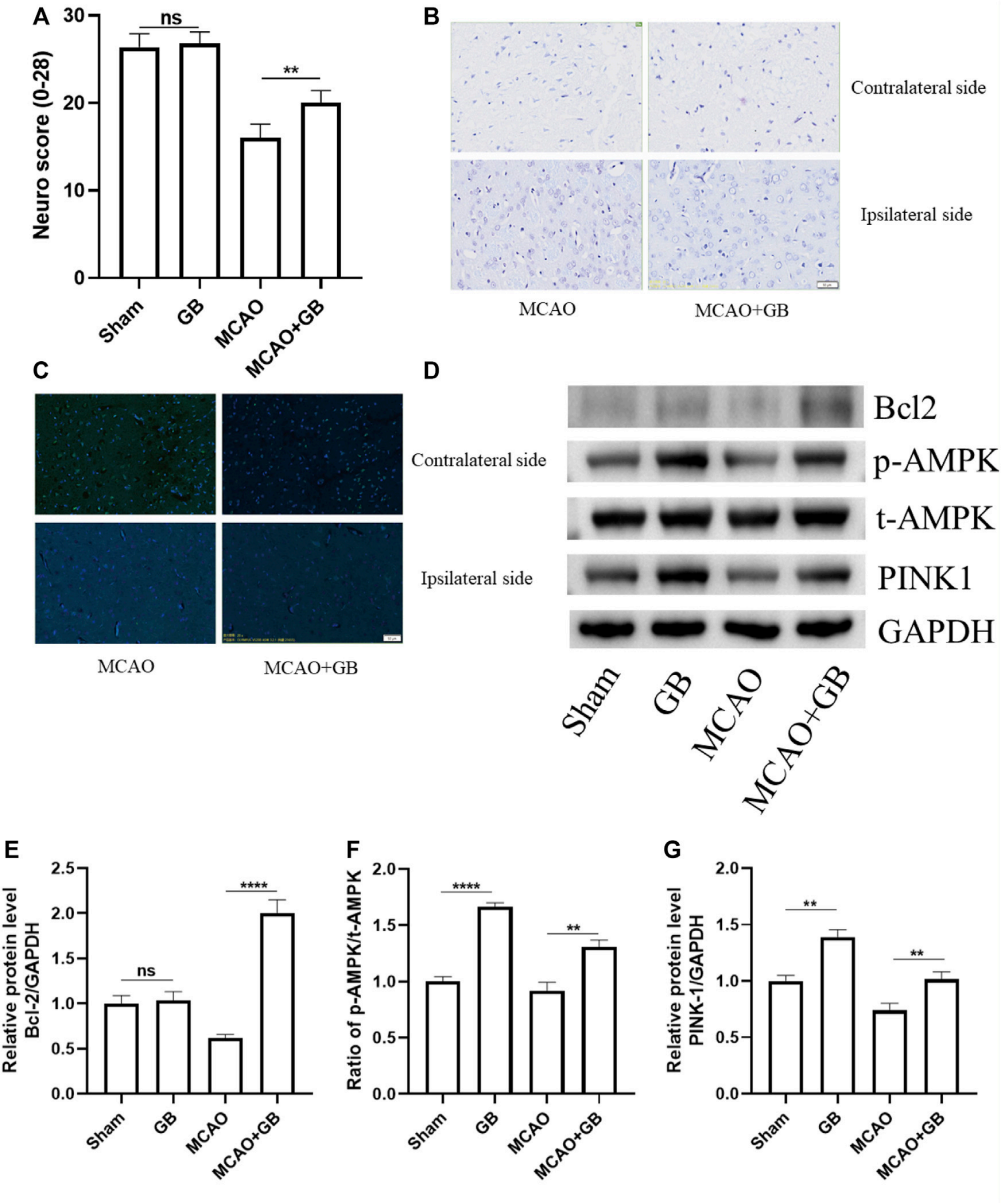
**(A)** The 28-point composite scoring system for neural behavior. Data are expressed as mean ± SEM and evaluated by means of one-way ANOVA (*n* = 6 per cohort). **(B)** Images of Nissl staining in the dorsal striatum following mild focal ischemia. Scale bar, 50 μm. **(C)** Images of the TUNEL staining in the dorsal striatum following mild focal ischemia. Scale bar, 50 μm. **(D–G)** Representative western blots of Bcl-2, AMPK, and PINK1 in rats exposed to MCAO treatment. (ns, no significant difference, **p < 0.01, ****p < 0.0001).

We utilized a 28-point composite neuro score to validate the neuroprotective effect of GB on sensorimotor dysfunction in rats following MCAO. We observed that there was a decrease in the scores of MCAO rats as opposed to the sham-operated controls, whereas neuronal loss and apoptosis showed an evident elevation in scores. It was also observed that GB treatment significantly reversed these changes (Figures 1A–C). The results of the western blot assay indicated that the decreased neuronal apoptosis in GB-treated MCAO rats was accompanied by an elevation in PINK1 and p-AMPK levels (Figures 1D–G). These results suggest that GB treatment could alleviate sensorimotor dysfunction following MCAO and could be AMPK/PINK1-dependent.

### Ginkgolide B Treatment Decreased Oxygen and Glucose Deprivation-R-Induced SH-SY5Y Apoptosis and was AMPK-Dependent

**FIGURE 2:**
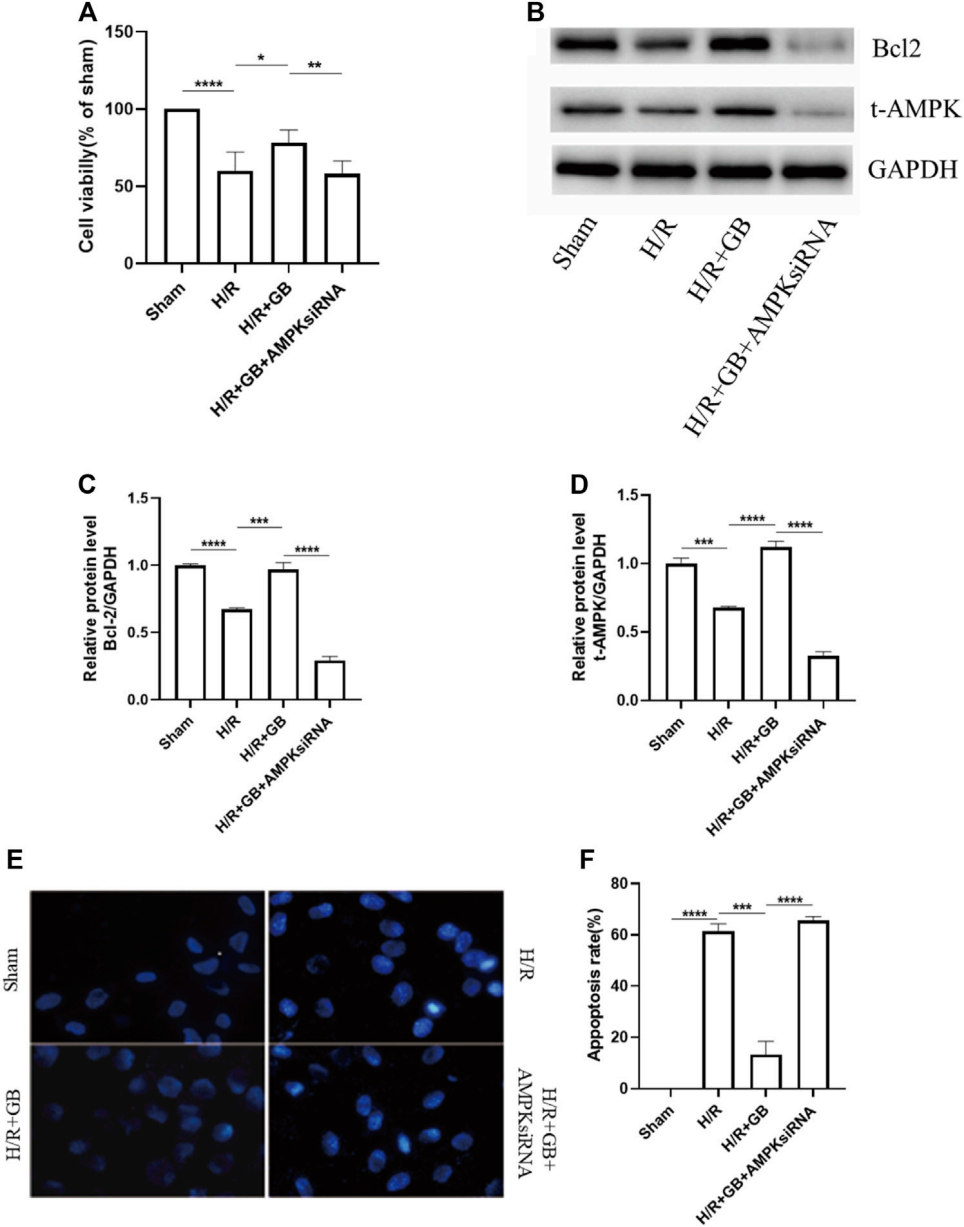
**(A)** MTT assay was utilized to evaluate cell viability. Data are expressed as mean ± SEM and are representative of 3 separate experimentations. **(B–D)** Western blot images of Bcl-2, AMPK in SH-SY5Y cells subjected to H/R and treated with or without GB. **(E,F)** Hoechst 33342 staining representative images **(E)**. Bar graphs illustrating the apoptosis rate of the cells subjected to various treatments **(F)**. Data are expressed as mean ± SEM and represent 3 distinct experiments. (***p < 0.001, ****p < 0.0001).

We used SH-SY5Y human neuroblastoma cells that had been cultured under the OGD-R circumstance as a model of ischemia-reperfusion damage for the purpose of investigating the protection impact of GB-related on neuronal cell death following ischemia. As shown in Figure 2A, there was a remarkably decreased viability in OGD-R treated cells as evaluated by the MTT assay. However, it was observed that GB treatment significantly reversed this decrease. AMPKα1/α2 siRNA effectively decreased AMPKα protein levels (Figures 2B–D) and led to a significant decrease in cell viability of GB-treated cells under OGD-R conditions. Similar results were found in SH-SY5Y cell apoptosis, as evidenced by the results of Hoechst 33342 staining (Figures 2E,F). These results suggest that GB treatment decreased SH-SY5Y cells apoptosis induced by OGD-R and was AMPK-dependent.

### Ginkgolide B Improved Oxygen and Glucose Deprivation-R-Induced SH-SY5Y Mitochondrial Damage and was AMPK-Dependent

**FIGURE 3:**
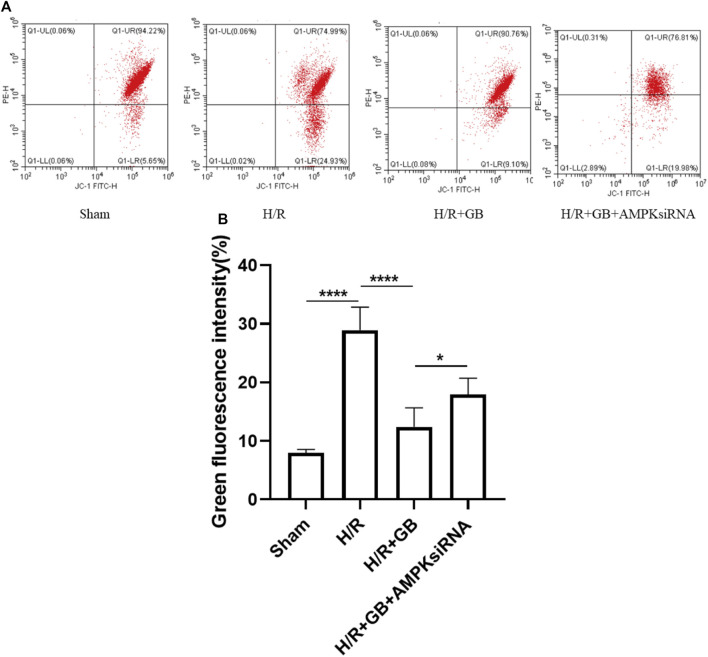
MMP measured *via* FACS **(A)**. Bar graphs showing the ratio of green/red rate in the cells subjected to various treatments **(B)**. Data are expressed as the mean ± SEM and represent 3 distinct experiments. (*p < 0.05, ****p < 0.0001).

The mitochondrial membrane potential (MMP) is an index that can be used to measure the extent of mitochondrial damage. The protective properties of GB in relation to mitochondrial damage were explored in SH-SY5Y cells following ischemia, as evaluated through JC-1 staining. The intensity of green fluorescence was substantially elevated following OGD-R treatment, demonstrating that the MMP of cells was attenuated. However, this decreasing trend was reversed upon treatment with GB. AMPK knockdown using AMPKα1/α2 siRNA abrogated the GB treatment-induced improvement of MMP in H/R-treated cells (Figure 3). These findings illustrated that GB could perform a function in reducing the mitochondrial damage response to OGD-R stress and that AMPK performs a critical function in this process. These results also indicated that the OGD-R-stimulated neuronal apoptosis was dependent on the mitochondrial apoptosis pathway. The level of Bcl-2 expression in OGD-R treated cells confirmed this finding.

### Ginkgolide B Upregulates the Expression of PINK1 in Response to Ischemia and was AMPK-Dependent

**FIGURE 4:**
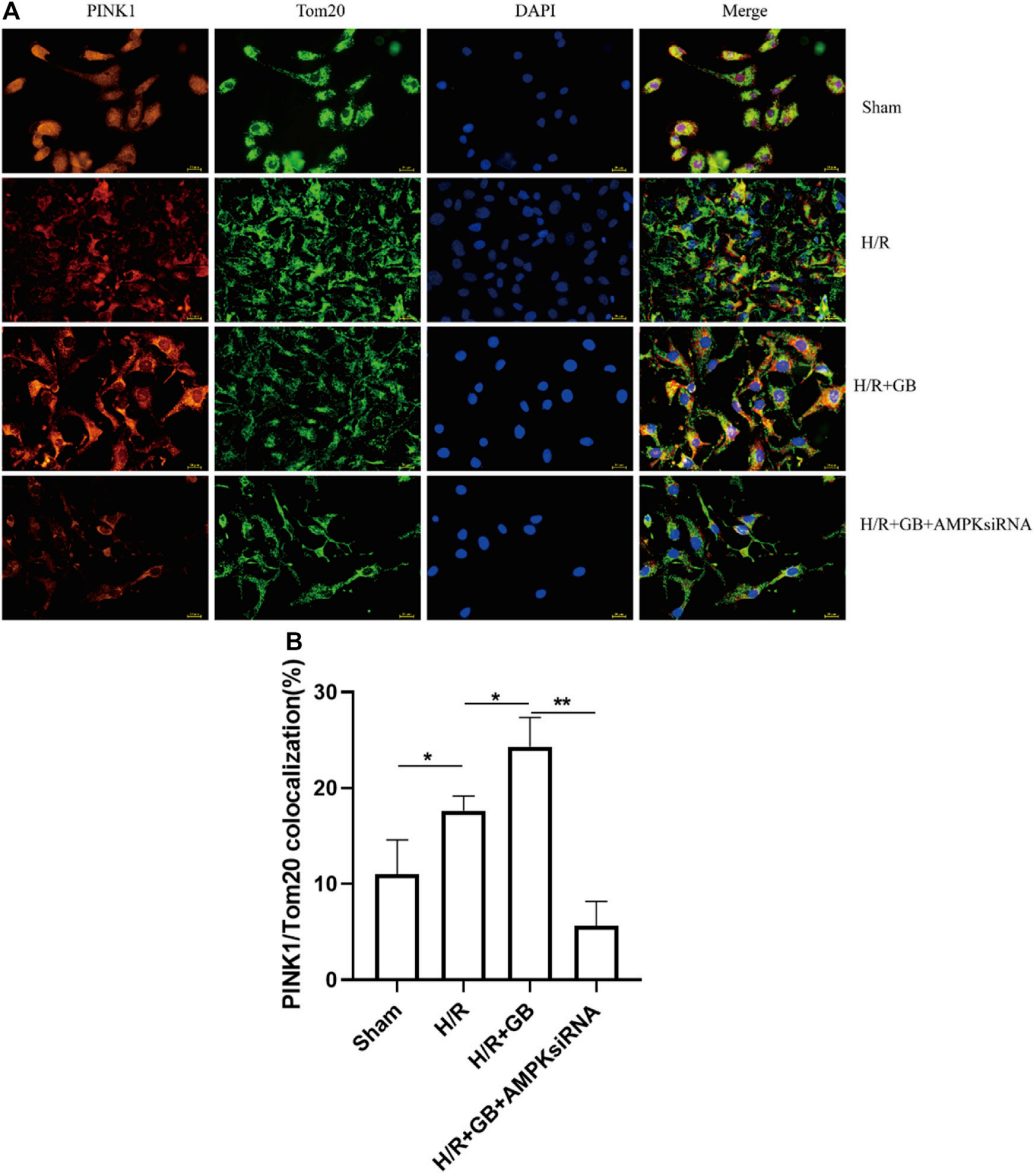
Representative images of PINK1 located on the outer mitochondrial membrane **(A)**. Bar graphs showing the ratio of PINK1/Tom20 co-localization in the cells subjected to various treatments **(B)**. Data are expressed as mean ± SEM and represent 3 separate experiments. (*p < 0.05, **p < 0.01).

The stable expression of PINK1 on the outer mitochondrial membrane performs a critical function in reducing mitochondrial damage. To explore the effect of GB treatment on the mitochondrial damage in SH-SY5Y cells following ischemia, the localization of PINK1 on the outer mitochondrial membrane in OGD-R cells was evaluated upon treatment with GB, as evidenced by the results of the double immunofluorescence staining. As shown in Figure 4, co-localization was slightly enhanced in cells treated with OGD-R compared to the sham control and was enhanced upon GB treatment. Moreover, this enhancing trend was abrogated upon treatment with AMPK siRNA. These results suggest that the increased PINK1 expression in the outer mitochondrial membrane upon GB treatment was AMPK-dependent.

## Discussion

Rat focal cerebral ischemia models induced *via* transient middle cerebral artery occlusion (t-MCAO) could produce a reliable infarct that strongly resembles human ischemic stroke (Fluri et al., 2015). This model was then widely used in studies focusing on ischemic pathophysiological processes and related neuroprotective strategies (Shah et al., 2019). In the present research, we examined the protection ability of GB against stroke and its specific mechanism using a rat t-MCAO model. It was observed that the neuroprotective functions of GB in MCAO rats resulted in a decrease in neuronal apoptosis, which was associated with the upregulated expression of PINK1 that was dependent on the activation of the AMPK pathway.

Neuronal apoptosis is a major pathological process following ischemia, and complex mechanisms are responsible for this mechanism. In the present research, we focused on exploring the neuroprotective effect of Ginkgolide B, a widely used compound in treating ischemic stroke, in relation to neuronal apoptosis. In MCAO rats, GB treatment significantly decreased neuronal loss and apoptosis and was accompanied by increased PINK1 expression and AMPK activation. These results suggest a potential novel link between GB and a mitochondrial damage response to cerebral ischemia. To delve into the underlying mechanism, SH-SY5Y human neuroblastoma cells that had been cultured based on the OGD-R circumstance were used to construct a model of ischemia-reperfusion damage (Miglio et al., 2004; Fallarini et al., 2009). Using this *in vitro* model, we showed that GB reduced mitochondrial damage by upregulating the expression of PINK1 in the outer mitochondrial membrane and that the observed upregulation of PINK1 prevents mitochondrial apoptosis. Meanwhile, to prevent the non-specific impacts of pharmacologic approaches with regard to the correlation between AMPK signaling pathways and GB, a genetic approach using AMPKα1/α2 siRNA was employed to silence AMPK. The results demonstrated that the protective function of GB on mitochondrial damage and apoptosis is dependent on AMPK activation. As far as we know, the present research is the first investigation to explore the role of GB on mitochondrial apoptosis in cerebral ischemia.

**FIGURE 5:**
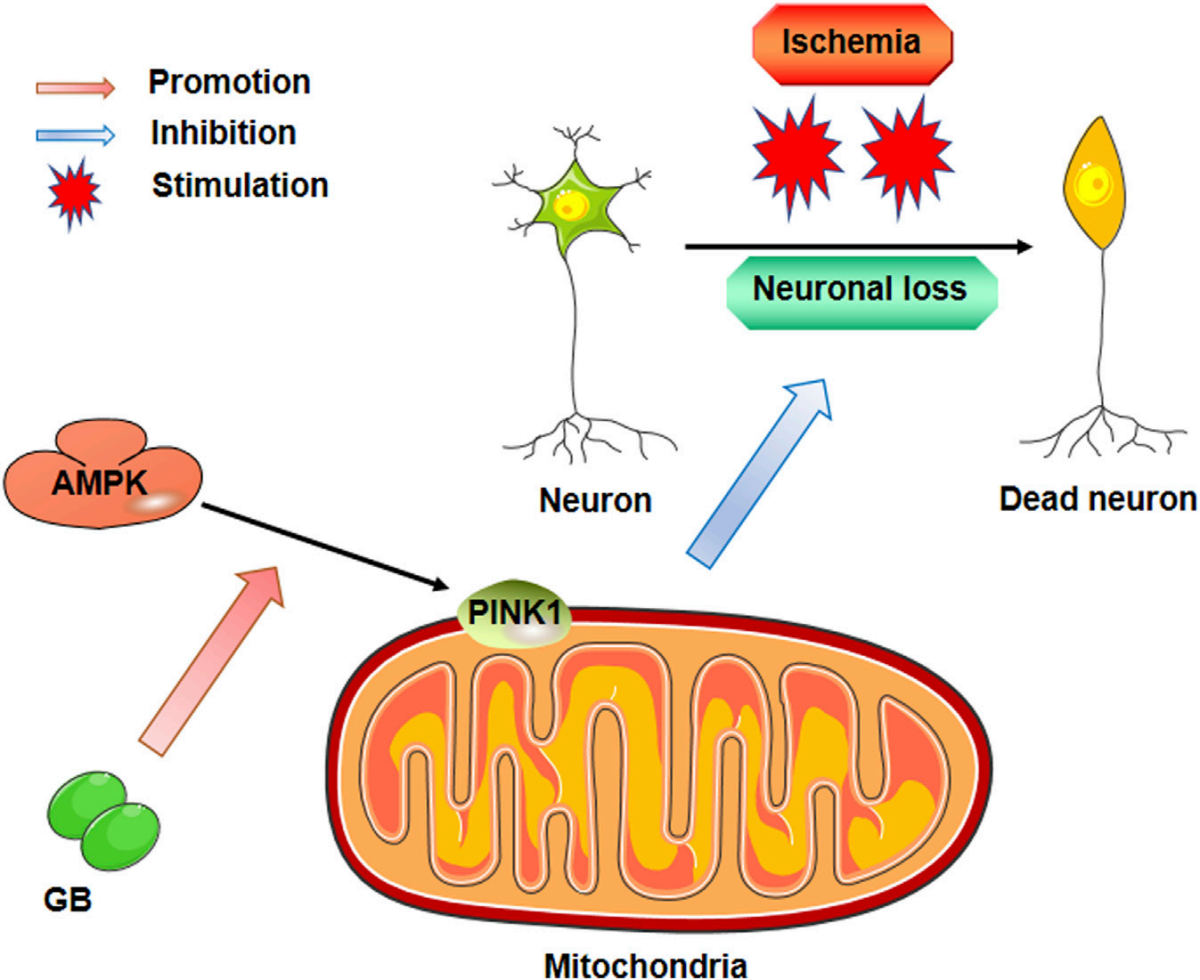
Pharmacological mechanism of Ginkgolide B against neuronal loss post ischemic stroke.

GB has been widely used to treat stroke (Chi et al., 2015; Feng et al., 2019) because of its anti-inflammatory (Gu et al., 2012; Shu et al., 2016; Li et al., 2020), metabolic, and neurotrophic effects (Nabavi et al., 2015; Yu et al., 2018; Xin et al., 2020; Yang et al., 2021). However, in clinical practice, neuro progresses to irreversible ischemic injury after stroke occlusion within a few minutes. The substances released from damaged nerve cells in such a short-time window induce the inflammatory response after stroke. As consequence, the anti-apoptotic properties of GB might have an integral function in stroke treatment although such a role is rarely reported. In the present research, the anti-apoptotic impact of GB on cerebral ischemia was confirmed by *in vivo* and *in vitro* experiments. Furthermore, our results corroborate that the AMPK pathway performs a key function in mitochondrial function and apoptosis (Figure 5). In summary, our study provided a novel possibility for stroke treatment that needs to be further studied.

## Data Availability

The original contributions presented in the study are included in the article/supplementary material, further inquiries can be directed to the corresponding authors.
